# Effect of Exercise and Calorie Restriction on Tissue Acylcarnitines, Tissue Desaturase Indices, and Fat Accumulation in Diet-Induced Obese Rats

**DOI:** 10.1038/srep26445

**Published:** 2016-05-20

**Authors:** Venkatesh Gopalan, Navin Michael, Seigo Ishino, Swee Shean Lee, Adonsia Yating Yang, K. N. Bhanu Prakash, Jadegoud Yaligar, Suresh Anand Sadananthan, Manami Kaneko, Zhihong Zhou, Yoshinori Satomi, Megumi Hirayama, Hidenori Kamiguchi, Bin Zhu, Takashi Horiguchi, Tomoyuki Nishimoto, S. Sendhil Velan

**Affiliations:** 1 Laboratory of Molecular Imaging, Singapore Bio Imaging Consortium, Agency for Science Technology and Research (A^*^STAR), Singapore; 2 Singapore Institute for Clinical Sciences, Agency for Science Technology and Research (A^*^STAR), Singapore; 3 Takeda Pharmaceutical Company Limited, Japan; 4 Takeda Singapore Pte Ltd., Singapore; 5 Molecular Imaging Centre, National Institute of Radiological Sciences, Chiba, Japan

## Abstract

Both exercise and calorie restriction interventions have been recommended for inducing weight-loss in obese states. However, there is conflicting evidence on their relative benefits for metabolic health and insulin sensitivity. This study seeks to evaluate the differential effects of the two interventions on fat mobilization, fat metabolism, and insulin sensitivity in diet-induced obese animal models. After 4 months of *ad libitum* high fat diet feeding, 35 male Fischer F344 rats were grouped (n = 7 per cohort) into sedentary control (CON), exercise once a day (EX1), exercise twice a day (EX2), 15% calorie restriction (CR1) and 30% calorie restriction (CR2) cohorts. Interventions were carried out over a 4-week period. We found elevated hepatic and muscle long chain acylcarnitines with both exercise and calorie restriction, and a positive association between hepatic long chain acylcarnitines and insulin sensitivity in the pooled cohort. Our result suggests that long chain acylcarnitines may not indicate incomplete fat oxidation in weight loss interventions. Calorie restriction was found to be more effective than exercise in reducing body weight. Exercise, on the other hand, was more effective in reducing adipose depots and muscle triglycerides, favorably altering muscle/liver desaturase activity and improving insulin sensitivity.

Obesity is a major risk factor for several cardio-metabolic diseases^1^,^2^. A sedentary lifestyle and a gradual shift towards a diet characterized by copious amounts of fat and refined carbohydrates have been the driving factors behind the global obesity pandemic^3^,^4^,^5^. A high-fat diet has been known to induce weight gain and insulin resistance. The lipid overload associated with high fat diet (HFD) and increased adiposity exposes the liver and skeletal muscle to increased fat trafficking. Fatty acids can enter either the oxidation or the esterification pathways. Interestingly, metabolic intermediates in both pathways have been linked to insulin resistance. Once fatty acids are taken up by hepatocytes or myocytes, stearoyl coenzyme desaturases (SCD) play a major role in determining their metabolic fates. The SCD enzyme converts saturated fatty acids to monounsaturated fatty acids, which are the preferred substrates for esterification. While triacylglycerols are metabolically inert, lipotoxic intermediates of esterification like long chain fatty acyl-CoAs, diacylgycerols and ceramides have been known to disrupt the insulin signaling pathways^6^. Polyunsaturated essential fatty acids taken up by the cells undergo desaturation by Δ^5^ desaturase (D5D) and Δ^6^ desaturase (D6D) enzymes to form long chain polyunsaturated fatty acids, which are required for increasing the cell membrane fluidity in insulin sensitive tissues. High fat diets, particularly those rich in saturated fatty acids, result in increased SCD and D6D activity and reduced D5D activity. These alterations in fatty acid desaturase activity have been associated with increased insulin resistance and type 2 diabetes mellitus (T2DM)^7^,^8^,^9^,^10^,^11^,^12^. Obesity and T2DM have also been characterized by elevated levels of serum long chain acylcarnitines^13^. Fatty acyl-CoAs are transported to the mitochondria for β-oxidation by first converting them to acylcarnitines. The mismatch between a high β-oxidative flux and low tricarboxylic acid (TCA) cycle flux in conditions of lipid overload was hypothesized to result in incomplete fat oxidation and an increased mitochondrial and cytosolic accumulation of long chain acylcarnitines^14^. It was initially suggested that mitochondrial oxidative phosphorylation genes are downregulated in response to chronic high fat diet feeding^15^, which seems to support the above hypothesis. However, this has been contested by more recent works which report that high fat diets are associated with elevated fat oxidation, mitochondrial oxidative enzyme activity, and mitochondrial biogenesis^16^. Either way, in resting state the energy requirements of the cellular housekeeping operations which determine the TCA cycle flux are always much lower than the maximal β-oxidative capacity^17^ and β-oxidative flux can be expected to be in excess of the TCA cycle flux to account for fluctuating energy requirements^18^. Computational modelling of the β-oxidation pathway suggests that, in the presence of excess long chain acyl-CoA precursors, the substrate competition between long chain acyl-CoAs and downstream short chain acyl-CoA products for acyl-CoA dehydrogenases may overload the β-oxidative pathway, resulting in reduced flux and increased accumulation of long chain acylcarnitines^19^. Long chain acylcarnitines have been associated with insulin resistance, although the precise mechanism is not yet clear^14^.

Exercise and calorie restriction are the two most commonly used lifestyle intervention for counteracting obesity. While the role of negative energy balance in inducing weight loss is well known, there is conflicting evidence on the relative benefits of the two strategies for improving metabolic health and insulin sensitivity^19^,^20^,^21^,^22^,^23^,^24^,^25^. In particular, the differential effects of exercise and calorie restriction on fat mobilization, fatty acid metabolism in liver and muscle, and insulin sensitivity in obese states are not well characterized. The goal of this study is to evaluate the relative effects of varied levels of exercise (exercise once a day (EX1), exercise twice a day (EX2) and calorie restriction (15% calorie restriction (CR1) and 30% calorie restriction (CR2) interventions on triglyceride accumulation, long chain acylcarnitine accumulation and desaturase activity in muscle and liver, abdominal fat depots, skeletal muscle mass, and insulin sensitivity in diet-induced obese Fischer F344 rats compared to sedentary controls (CON).

## Results

**Figure 1:**
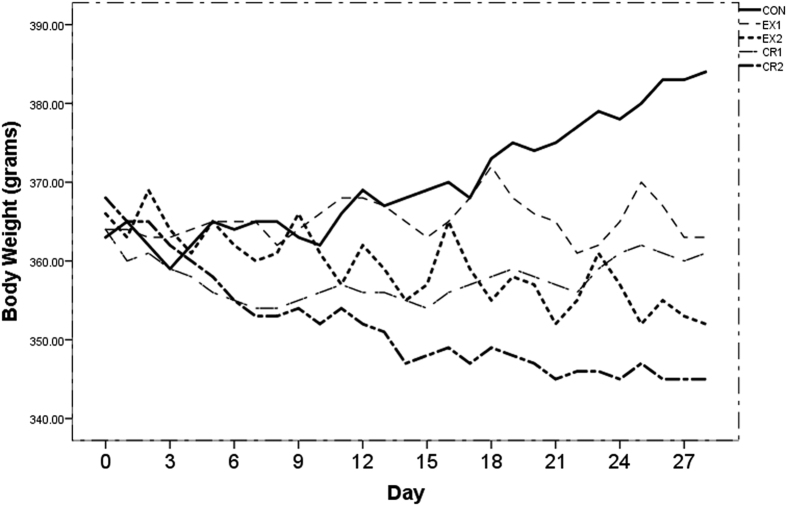
Body weight curves for different groups during the 28 days of intervention period for control, exercise and calorie restriction groups.

All animals weighed between 360 g and 370 g after 4 months of *ad libitum* HFD feeding. One-way ANOVA of the baseline measurements of body weight, metabolic parameters, adipose depots (subcutaneous (SAT) and visceral adipose tissue (VAT)) and ectopic fat, shown in Table 1, did not reveal any statistically significant differences, except for MRI based VAT volumes^26^. The change in body weight over the 4-week intervention period is shown in Fig. 1. Table 2 shows the post-intervention metabolic measurements including the mean daily food intake and calorie deficits during the 28-day intervention. Differences in the plasma triglycerides, glucose, insulin, and insulin sensitivity after intervention were not statistically significant. The differential practical effects of the interventions with respect to the sedentary controls were assessed using the Glass’s ∆ effect size measure. The effect size measures of the different interventions for body weight, adipose depots, ectopic fat, and metabolic parameters are shown in Table 3. The following rule of thumb was used for evaluating the magnitude of the effect: small effect (∆ ≥ 0.2), moderate effect (∆ ≥ 0.5) and large effect (∆ ≥ 0.8). The results in Table 3 indicate a large reduction in body weight, leptin, and intra-hepatic lipid for all four interventions, when compared to the control group. MRI based SAT and VAT measurements^26^ also indicate a large reduction in the CR2, EX1 and EX2 cohort, but only a moderate reduction in the CR1 cohort. While the calorie restriction interventions had a larger effect on the body weight, exercise interventions had a greater effect on the SAT and VAT volumes. The EX1 and EX2 cohorts also showed a large increase in the tibia muscle weight, suggesting an increase in the skeletal muscle mass, despite reduced body weight. Plasma triglycerides were reduced in the calorie restriction cohorts and elevated in exercise cohorts. There was a moderate reduction in muscle triglycerides in the calorie restriction groups and a large reduction in the exercise groups. In spite of the lower muscle total triglycerides levels, intramyocellular lipid (IMCL) levels were elevated in the exercise cohorts. Calorie restriction resulted in moderate improvements in insulin sensitivity, while exercise resulted in a large improvement.

The effect of the interventions on the fatty acid desaturase in the liver and muscle are shown in Table 4. There were moderate to large reductions in SCD16 and D6D indices and a large increase in D5D index in the tibialis muscle for all the intervention cohorts. There was no significant difference in the hepatic SCD16 index in the CR1 cohort. Large increase in the hepatic SCD16 index was observed in the CR2, EX1 and EX2 cohorts. Similar to the muscle, there was a moderate to large reduction in liver D6D index and a large increase in liver D5D index in all the intervention cohorts.

The effect sizes of the interventions on the liver and muscle acylcarnitines are shown in Table 5. Free carnitine in liver and muscle (AC C0) levels were elevated in all intervention cohorts, with CR1, CR2 and EX1 showing a moderate effect, while EX2 showed a small effect. Muscle free carnitine (AC C0) was also moderately increased in the calorie restriction cohorts, while EX1 and EX2 cohorts showed a large and small increase, respectively. Surprisingly, long chain acyl carnitines, AC C16:0, AC C18:0 and AC C18:1 were not reduced in both muscle and liver, as expected. On the other hand, there were small to large increases in the liver and muscle long chain acylcarnitines in the CR1, EX1 and EX2 cohorts, except for muscle AC16:0 levels in the EX1 cohort. There were no practically significant effects on long chain acyl carnitines in the CR2 cohort except for a small increase in muscle AC 18:0. The skeletal muscle ketogenic marker AC C4-OH was reduced in all intervention cohorts with CR1 and CR2 showing a moderate effect, and EX1 and EX2 cohorts showing a small and large effect respectively.

The results of pooled cohort correlation analysis are shown in Table 6. The percentage body fat showed a strong positive association with adipose depot volumes, leptin, intrahepatic lipid (IHL), SCD16 and D6D indices in liver and muscle and a strong negative association with insulin sensitivity and D5D indices in liver and muscle. Insulin sensitivity showed a strong positive association with long chain acylcarnitines in liver, but not in muscle. It was negatively associated with liver and muscle SCD16 indices, muscle D6D index, adipose depots and IHL, and positively associated with liver D5D index. IMCL showed a moderate positive association with muscle long chain acylcarnitines, while IHL did not show any similar association with the hepatic long chain acylcarnitines.

## Discussion

Conventionally, the amount of weight loss is used to evaluate the effectiveness of an anti-obesity intervention. Calorie restriction and exercise interventions resulted in large reductions in the body weight of the obese rats with respect to the CON cohort, over a 4-week period. Increased exercise duration and calorie restriction resulted in an increased effect on weight loss. However, evaluating the efficacy of the interventions only by weight loss may mask the changes observed in body composition and fat metabolism which can influence metabolic health. While CR1 and CR2 had a higher effect on weight loss, the EX1 and EX2 cohorts were more effective in increasing the tibialis muscle wet weight, and reducing the adipose depot volumes, tibialis muscle triglycerides, circulating leptin levels, and % body fat. These results suggest that exercise is more effective for mobilizing the fat mass than calorie restriction. Loss of lean mass may account for a greater proportion of the weight loss in the calorie restriction interventions than in exercise interventions, which can have adverse metabolic effects. Loss of lean mass during calorie restriction may be related to the reduced protein intake, and increased protein catabolism during periods of high energy stress^27^.

We tried to estimate the energy cost of the different interventions, since this influences the ability to induce weight loss. The served food portions in the CON, EX1 and EX2 cohorts were identical, but the mean food intake of the EX1 and EX2 cohorts were lower than that of the CON cohort (mean calorie deficit of 4.13 kcal/day and 4.63 kcal/day respectively), possibly due to exercise-induced hypophagia. Based on oxygen cost of treadmill exercise as reported in literature^28^, we obtained an estimated daily exercise-induced energy expenditure of 1.04 kcal and 2.08 kcal in the EX1 and EX2 cohort, respectively (see supplementary information). The combined energy costs of treadmill running and hypophagia-induced calorie deficit in the exercise cohort are still lower than the dietary calorie deficits in the calorie restriction cohorts, and may contribute to the higher weight loss with calorie restriction.

The behaviour of ectopic fat mobilization in the different cohorts was very different from the mobilization of fat in adipose tissue depots. The post-intervention ^1^H MRS liver fat fraction was reduced in all the intervention cohorts. Increasing the duration of exercise and degree of calorie restriction resulted in greater reduction in liver fat fraction. We found no effect of calorie restriction on IMCL levels. There was a small increase in IMCL levels in the EX1 and the large increase in EX2 when compared to the CON cohort, in spite of a reduction in the total tibialis triglycerides. This can be attributed to the effect of exercise training on IMCL. Exercise training has been associated with a reduction in subsarcolemmal IMCL droplets, but an increase in intermyofibrillar IMCL droplets in direct contact with the mitochondria^29^. IMCL droplets close to the mitochondria may serve as a readily available fuel source during exercise. Other weight loss interventions based on exercise, calorie restriction, or a combination of both in obese/overweight humans have reported a reduction in weight, liver fat and visceral fat, and an improvement in metabolic health with no change in the IMCL levels^30^,^31^.

High fat diets, obesity and insulin resistance states have been linked to increased SCD and D6D activity and reduced D5D activity^7^,^9^,^12^. The D6D and D5D are key enzymes involved in the synthesis of long chain polyunsaturated fatty acids derived essential fatty acids. The D6D and D5D activities are regulated in the opposite direction since the elongated product of the D6D enzyme acts as the precursor for the D5D enzyme. All the interventions showed moderate to large reductions in the liver and muscle D6D indices, and a large increase in the D5D index. The products of the D5D enzyme, arachidonic acid, eicosapentaenoic acid, and docosahexaenoic acid, are essential for improving the cell membrane fluidity, which results in an increase in the number of insulin receptors and increased sensitivity to insulin^8^. They are also important for the synthesis of cellular signaling molecules, prostaglandins and resolvins which have a number of pro-inflammatory and anti-inflammatory functions^8^. Our results indicate large reductions in the liver and muscle SCD16 indices in all the intervention cohorts. The reduced SCD16 index suggests that both the interventions are capable of increasing the intracellular fat partitioning to oxidation rather than esterification. Reduced partitioning towards fat esterification is associated with a reduced accumulation of lipotoxic lipid intermediaries like long chain acyl-CoA, diacylglycerols, ceramides, which have been linked to insulin resistance^6^.

As opposed to the lipotoxicity based theories linking intermediates of esterification to disrupted insulin signaling, Koves *et al.*^14^ had proposed that high fat diets and obesity are linked to elevated rates of incomplete fat oxidation, which results in the intracellular accumulation of long chain acylcarnitines. It was hypothesized that the accumulation of long chain acylcarnitines was linked to insulin resistance, although the mechanism is not yet clear. The incomplete fat oxidation hypothesis has been supported by studies showing elevated long chain acyl carnitines in insulin resistant, obese subjects and T2DM patients^13^,^32^. A 10-week aerobic exercise intervention study in humans^27^ and a 2-week free wheel running based intervention in HFD fed obese mice^33^ were both shown to result in reduced long chain acylcarnitines. In light of these observations, we had hypothesized that a 4-week exercise or calorie restriction intervention would reduce the long chain acylcarnitines levels when compared to sedentary HFD fed rats, either by reducing the mitochondrial lipid overload or by enhancing complete oxidation of fatty acids to CO_2_. Contrary to our hypothesis, we found that liver and muscle long chain acylcarnitines were elevated in all the intervention cohorts except CR2. However, our results confirm the observations of a number of weight loss studies which report increased long chain acylcarnitines after weight loss. Calorie restriction in obese humans was accompanied by elevated fasting long chain acylcarnitines^34^,^35^,^36^ and total acylcarnitines^37^. Weight reduction in young insulin resistant children of parents with T2DM was reported to result in improved glucose tolerance, without any statistically significant changes in long chain acylcarnitines^38^. However, on calculating the effect sizes from the raw values reported in the above work, we found a practically significant increase in long chain acylcarnitines with a Glass’s ∆ of 0.72. Voluntary wheel running in middle aged rats was also found to result in 2-fold to 3-fold increase in long chain acyl carnitines in the plantaris muscle^39^.

Accumulation of long chain acylcarnitines in conditions of obesity and lipid stress has been associated with the depletion of the intracellular free carnitine pool^40^. In the current study, we found that elevated long chain acylcarnitines after the weight loss interventions were not accompanied by a depletion of the intracellular free carnitine pool. In fact, there were small to large increases in muscle and liver free carnitines in all the intervention cohorts relative to the control cohort. Muscle β-OH buturylcarnitine (AC C4-OH) is a marker for ketogenesis, which is usually considered to be a hepatic program^41^. Within the muscle, it has been hypothesized that *de novo* ketogenesis could provide an outlet for acetyl-CoA produced in excess of TCA cycle requirement, in states of lipid overload^42^. We found that the intervention cohorts showed small to large reduction in muscle AC C4-OH, which suggests that interventions did not lead to production of acetyl-CoA in excess of the TCA flux. The elevated free carnitine levels and reduced muscle AC C4-OH suggest that in spite of elevated long chain acylcarnitines, the weight loss intervention cohorts do not fully recapitulate all the defects associated with lipid stress and overloaded mitochondria in obese and insulin resistant states. We also note that SCD index was reduced in all the intervention cohorts. SCD deficiency in liver^43^ and muscle^44^ in rodent models has been associated with increased phosphorylation of 5′ adenosine monophosphate-activated protein kinase (AMPK) and increased carnitine palmitoyl transferase1 (CPT1) activity, which results in increased transport of fatty acyl CoAs to the mitochondria for β-oxidation. Therefore, increased acylcarnitines in the intervention cohorts may just reflect an elevated mitochondrial β-oxidative flux, and not incomplete fat oxidation. The lack of a practically significant increase in liver and muscle long chain acylcarnitines (except muscle AC C18:0) in the CR2 cohort, unlike the CR1, EX1 and EX2 cohorts may be most likely due to a sharp reduction in the supply of dietary fatty acids due to the 30% calorie restriction.

Differences in the post-intervention plasma measurements of triglycerides, glucose, insulin and insulin sensitivity were not statistically significant. This is likely due to the fact that, only a very large magnitude of intervention effect, similar to the effect of the intervention on fat depots, is likely to be statistically significant for such a small sample size (n = 7 per group). The plasma triglycerides also showed a high degree of variability in the exercise cohort, which may be partly influenced by the stress associated with forced treadmill exercise and electro-shock^45^. Analysis of the Glass’s ∆ effect size measures in Table 3 indicated that insulin sensitivity, as assessed by quantitative insulin sensitivity check index (QUICKI), was improved in all the intervention cohorts, with calorie restriction showing a moderate effect and exercise showing a large effect. This seems to suggest that exercise interventions were more effective in improving the metabolic health when compared to calorie restriction. However, we need to add the caveat that the insulin sensitivity measurements may be partly influended by the acute effects of exercise. The blood samples were withdrawn 19 and 27 hours after the last bout of exercise in the EX2 and EX1 cohort, respectively. QUICKI is more strongly influenced by hepatic insulin resistance than peripheral insulin resistance. Both peripheral and hepatic insulin sensitivity are improved acutely after exercise^46^,^47^,^48^,^49^. The improvement in peripheral insulin sensitivity lasts for 24–48 hours after the last bout^46^. Improved hepatic insulin sensitivity has been reported up to 8 hours after the last bout of exercise^48^ and whether these effects last for a longer duration is not clear.

The pooled cohort correlation analysis indicated that the % body fat showed a strong statistically significant association with both liver and muscle desaturase indices but not the liver and muscle acylcarnitines. This suggests that weight loss interventions that are more effective in reducing the % body fat may also be more effective in altering pathogenic changes in desaturase activity due to obesity and high fat diet. Insulin sensitivity assessment using QUICKI was negatively associated with % body fat, VAT and IHL, while the associations with SAT and IMCL were not statistically significant. SCD16 and D6D indices in the liver and muscle showed moderate to strong negative associations, while liver D5D showed a positive association with insulin sensitivity. These trends largely confirm the associations between insulin sensitivity and desaturase activity reported in literature. We found that after the weight loss intervention, insulin sensitivity was positively associated with hepatic long chain acylcarnitines. This is in contrast to earlier works^13^,^32^, which found a negative association between plasma acylcarnitines and insulin sensitivity in obese and glucose intolerant states. This suggests that elevated acylcarnitines may not reflect impaired fat oxidation in weight loss interventions. We found a positive association between liver SCD16 index and IHL. Increased SCD activity is associated with increased production of monounsaturated fatty acids which are the preferred substrates for esterification. The IHL showed a strong negative association with D5D (r = 0.8), which seems to confirm the negative association between long chain omega-3 fatty acids and IHL^50^. D5D and D6D enzymes are required for synthesizing long chain omega-3 fatty acids from essential fatty acids. Additionally, we found that IHL was also positively associated with muscle SCD16 and D6D indices and negatively with the muscle D5D index. IMCL was not related to any of the desaturase indices but was positively associated with the muscle long chain acylcarnitines. Plasma fatty acids are first cycled through the IMCL pool before entering the oxidative pool^51^. Thus, fatty acids obtained from IMCL have been suggested to be the immediate precursors of the fatty acids in skeletal muscle mitochondria, which might explain the above positive association between IMCL and muscle long chain acylcarnitines.

In conclusion, we found that 4-week of exercise and calorie restriction were both capable of inducing a large weight loss in HFD fed obese rats, when compared to sedentary controls. Calorie restriction was more effective than exercise in inducing weight loss, but exercise was more effective than calorie restriction in increasing muscle weight, reducing adipose depots and muscle triglycerides, and reversing the alteration in desaturase activity associated with a high fat diet. Exercise was also more effective in improving insulin sensitivity, although this could be partly influenced by the insulin sensitizing effects of the last bout of exercise. Contrary to our hypothesis, we also found the exercise and mild (15%) calorie restriction interventions were associated with elevated long chain acylcarnitines. However, there was no depletion of the free carnitine pool or elevation in muscle AC C4-OH in these cohorts, which normally accompanies long chain acylcarnitine accumulation associated with lipid overload. The improved insulin sensitivity in these cohorts suggests that elevated long chain acylcarnitines after weight loss may reflect elevated β-oxidative flux, and not incomplete fat oxidation.

## Materials and Methods

### Animals

All studies were conducted at Singapore Bioimaging Consortium (SBIC), Singapore. Male Fischer (F344) rats were obtained from CLEA-Japan, Tokyo, Japan. All *in vivo* experiments involving animals were approved by the Institutional Animal Care and Use Committee, Biological Research Centre of Agency for Science Technology And Research (A*STAR), Singapore. All methods were carried out in accordance with the approved guidelines. After 1-week of habituation, 35 rats (5 weeks of age) were fed with gamma-irradiated high fat diet (D12079B, Research Diet, Inc. New Brunswick, NJ) and sterile water *ad libitum* for 4 months. The daily food intake was monitored. The mean daily food intake of the combined cohort during the *ad libitum* feeding period was 14 g. The temperature was controlled at 23 °C, with a humidity range from 30–70% and a constant light-dark cycle (12 h: 12 h).

### Study protocol

For the 4-week intervention study, the diet-induced obese rats (DIO) were divided into five cohorts (n = 7, per cohort): CON, EX1, EX2, CR1 and CR2. To eliminate the confounding effect of changes in fat or macronutrient composition on fat mobilization and desaturase activity, the HFD composition was kept identical in all the cohorts during both the 4-month *ad libitum* feeding and the 4-week intervention periods. Between day 0 and day 27, the daily food portion for the CON, EX1 and EX2 cohorts was fixed at the mean *ad libitum* food intake value of 14 g, while calorie restriction in the CR1 and CR2 cohorts was performed by limiting the daily food portions to 85% and 70% of the mean food intake, respectively. The daily food intake was also monitored over the course of the 4-week intervention period. The rats in the EX1 and EX2 cohorts were made to run on a treadmill (model Exer-3/6, Columbus Instruments, OH) once a day (9 am) in the EX1 group and twice a day (9 am and 5 pm) in the EX2 group for 5 days per week. Each exercise session lasted for 30 minutes. To acclimatize the rats to treadmill running, treadmill speeds were set at 16 m/min on day 0, 18/min on day 1 and 20 m/min from day 2 to day 27. The control group (CON) did not undergo any weight loss intervention. Detailed methods are described below.

### Physiological measurements

Over the period of the interventions starting from day (0) to the endpoint of the study at day (28), body weight was measured on weekdays. Blood sampling was performed on day 0 and day 28 with a fasting period of 4 hours. Blood withdrawal did not exceed 0.5 ml and the amount of blood did not exceed a total of 7.5% of circulating volume per week, in accordance with internationally accepted guidelines^52^. Leptin and insulin values were measured using rat Leptin ELISA kit and an ultra-sensitive rat insulin ELISA kit (Crystal Chem, Inc. IL). Glucose measurements were done by Quest Laboratories Pte. Ltd. (Singapore) using an ADVIA Chemistry glucose hexokinase_3 (GLUH_3) reagent kit (Siemens Healthcare Diagnostics Inc. NY). Insulin sensitivity was estimated using the quantitative insulin sensitivity check index (QUICKI)^53^ which is defined as 1/(log(Fasting Glucose (mg/dL)) + log(Fasting Insulin (μU/mL)). A conversion factor of 1 ng/ml insulin = 23.4 μU/ml was used for calculating QUICKI^54^.

### Magnetic resonance imaging and spectroscopy

^1^H MRS and MRI measurements were performed 4 days before the start of the intervention taking into account the required recovery time from anesthesia effects. Post-intervention measurements were made after blood sampling on day 28. Imaging was performed using a 7 Tesla Bruker ClinScan with a volume transmit/receive coil along with surface receive coils in liver, abdomen and skeletal muscle. The rats were anesthetized using Attane^TM^ Isoflurane (Bomac Pty Ltd) (O_2_; 400 ml/min, Air; 1.0 L/min) during the scans.

Localized PRESS experiments were performed on liver and skeletal muscle with a voxel size of 64 mm^3^ and 27 mm^3^, respectively with TR = 4.0 s, TE = 13 ms. MR spectra were acquired from the right lobe of the liver (with an external trigger for motion compensation) and tibialis posterior region of the skeletal muscle of left hind limb. The voxel positions in the pre- and post-intervention scans were carefully matched using the localizer images. A water unsuppressed spectrum (4 averages) and a water suppressed spectrum (128 averages) were acquired from each voxel. Abdominal imaging was performed using a T_2_-weighted spin echo sequence with a field of view (FOV) of 65 × 65 mm^2^ and matrix size of 320 × 320. Abdomen images in the coronal plane were used as anatomical reference and transverse images from L1 to L5 vertebrae of the spine were acquired.

### MR data processing

The quantitation of the liver and muscle MR spectra was performed using Linear Combination Model (LCModel)^55^. The unsuppressed water signal was employed for eddy current correction. The IHL content was estimated using the liver fat fraction, which was calculated as the ratio of the lipid peak area to the sum of lipid and unsuppressed water peak areas. The methyl and methylene peaks arising from the hepatic triglycerides were used to calculate the total lipid peak area. The water and lipid peak areas were corrected for T_2_ losses before calculating the fat fraction. The water and lipid T_2_ values were obtained by repeating the MRS acquisitions at 6 different echo times and performing an exponential fit to the peak areas^56^. The tibialis muscle IMCL content was measured as the ratio of the IMCL methylene peak normalized by the creatine 3.0 ppm peak^57^. Segmentation of visceral and subcutaneous fat was performed using a hybrid segmentation (Region-based active contours and Fuzzy C-means algorithm) method by an in-house developed MATLAB® program^26^.

### Tissue extraction and biochemical analysis

The rats were euthanized after terminal experiments (day 28). Tissue samples were taken from the tibialis muscle and liver, from the same locations where the voxels were placed during the *in vivo* MRS scans. Tissue samples were weighed and immediately frozen in liquid nitrogen and stored at −80 °C for triglyceride measurement and LC-MS analysis. The total triglyceride content in the tibialis muscle extracts was measured using a Lab Assay (TM) Triglyceride for Cell biology (Wako Pure Chemical Industries, Ltd., Japan). The gonadal, mesenteric, retroperitoneal, perirenal and subcutaneous fat pads were extracted and weighed. The ratio of the sum of the weights of the fat pads to the total body weight was recorded as the % body fat.

### LC-MS and estimation of desaturase indices

After thawing, liver and tibialis muscle were homogenized with 9-fold (v/w) of isopropanol, and then centrifuged by 15000 rpm for 5 min. The sample solution was applied to a high performance LC-MS analysis. Five μL of the sample solution was injected to the liquid chromatography system, for which Ultimate 3000 RSLC system (Thermo Fisher Scientific, San Jose, CA) was selected. Chromatographic separation was performed by a gradient elution on a reverse phase column, Xbridge C18 (2.5 μm, 2.1 × 50 mm, Waters, Milford, MA), under the column temperature of 60 °C. The solvent system consisted of 0.01% acetic acid-1 mM NH_3_-2 μM EDTA-2Na in distilled water (solvent A) and 0.001% acetic acid-0.2 mM NH_3_ in ethanol-isopropanol (3:2, v/v) (solvent B) with a flow rate of 0.5 mL/min. The following gradient program was applied to the chromatographic separation: 0–1 min, 1% solvent B; 1–1.2 min, 1 to 55% solvent B; 1.2–2.7 min, 55 to 75% solvent B; 2.7–3.5 min, 75 to 99% solvent B; 3.5–6 min, 99% solvent B; and 6–8 min, 1% solvent B. The eluate from the liquid chromatography system was directly introduced to electrospray ionization on a LTQ-Orbitrap XL mass spectrometer (Thermo Fisher Scientific, San Jose, CA). The mass spectrometry data were obtained by an Orbitrap with the resolution of 70,000 full width of half maximum (FWHM) by both positive and negative ionization mode. The raw data were extracted by Expressionist RefinerMS (Genedata AG, Basel, Switzerland). Free fatty acids and acylcarnitines were identified based on the accurate molecular mass and the retention time of liquid chromatography. The LC-MS estimates of the composition of the free fatty acid pool in the liver and muscle extracts were used to measure the desaturase and elongase enzyme activity, using the following product-to-precursor ratios: C16:1/C16:0 (SCD16), C18:3/C18:2 (D6D) and C20:4/C20:3 (D5D)^58^.

### Statistical analysis

Statistical analysis was performed using SPSS statistical software package (version 16.0 for Windows, SPSS Inc., Chicago, Illinois). One-way analysis of variance (ANOVA) was used to identify differences in the mean values of the baseline and post-intervention measurements. The relative practical effectiveness of the four interventions with respect to the control cohort was assessed using the Glass’s ∆ effect size measure, which is evaluated by standardizing the mean difference between the intervention and control cohort’s post-measurements by the standard deviation of the control cohort. The effect size gives a meaningful relative quantitative comparison of the practical/clinical effects of the intervention. The associations between the post-intervention measurements of percentage body fat, insulin sensitivity, ectopic fat, desaturase indices and tissue acylcarnitines in the pooled cohort were measured using Pearson correlation coefficients (r).

## Additional Information

**How to cite this article**: Gopalan, V. *et al.* Effect of Exercise and Calorie Restriction on Tissue Acylcarnitines, Tissue Desaturase Indices, and Fat Accumulation in Diet-Induced Obese Rats. *Sci. Rep.* **6**, 26445; doi: 10.1038/srep26445 (2016).

## Figures and Tables

**Table 1:** Baseline measurements (post 4 months of *ad libitum* HFD feeding) of body weight, adipose depots, ectopic fat and serum metabolic parameters (mean ± sd, P < 0.05 in bold).

Measurements/Cohort	CR1(N = 7)	CR2(N = 7)	EX1(N = 7)	EX2(N = 7)	CON(N = 7)	P-value
Triglycerides (mg/dL)	462.86 ± 118.23	507.00 ± 144.03	454.29 ± 79.71	501.43 ± 174.60	433.71 ± 100.49	0.790
Glucose (mg/dL)	176.57 ± 25.94	165.29 ± 13.39	164.43 ± 17.56	182.14 ± 19.36	156.57 ± 13.66	0.102
Insulin (ng/mL)	5.66 ± 2.01	4.30 ± 1.18	4.62 ± 0.58	4.89 ± 1.29	4.60 ± 0.88	0.360
QUICKI@	0.231 ± 0.011	0.238 ± 0.008	0.236 ± 0.004	0.232 ± 0.005	0.237 ± 0.003	0.248
Leptin (ng/mL)	26.67 ± 4.77	24.50 ± 6.94	22.76 ± 3.03	26.29 ± 7.44	22.09 ± 4.46	0.452
Body weight (g)	363.72 ± 4.63	368.46 ± 6.44	364.09 ± 7.12	365.75 ± 10.99	362.86 ± 5.64	0.634
Intrahepatic lipid#	26.79 ± 4.36	3.99	25.15 ± 2.84	25.48 ± 4.71	27.98 ± 5.60	0.764
Intramyocellular lipids$	1.21 ± 1.25	1.34 ± 0.54	1.13 ± 0.42	0.96 ± 0.36	1.08 ± 0.32	0.873
SAT (mm^3^)	474.21 ± 166.86	91.18	281.41 ± 86.40	442.26 ± 108.37	351.15 ± 218.11	0.128
VAT (mm^3^)	10668.97 ± 1031.15	10231.29 ± 711.94	9712.57 ± 997.59	10456.37 ± 836.31	9222.44 ± 1053.02	**0.048**

^#^Intrahepatic lipid was calculated by ratio of (Fat/(Fat + H_2_O))*100.

^$^Intramyocellular lipid = Intramyocellular lipid/total creatine.

^@^QUICKI = 1/(log(Fasting Glucose (mg/dL)) + log(Fasting Insulin (μU/mL)).

**Table 2:** Post-intervention measurements of body weight, adipose depots, ectopic fat and serum metabolic parameters, daily food intake and daily dietary calorie deficit during 28-day intervention (mean ± sd, P < 0.05 in bold).

Measurements/Cohort	CR1(N = 7)	CR2(N = 7)	EX1(N = 7)	EX2(N = 7)	CON(N = 7)	P-value
Triglycerides (mg/dL)	459.43 ± 76.22	424.71 ± 69.78	498.43 ± 142.39	563.14 ± 248.65	473.14 ± 56.04	0.435
Glucose (mg/dL)	158.93 ± 15.32	163.23 ± 19.25	164.71 ± 24.69	160.03 ± 21.34	158.83 ± 9.61	0.965
Insulin (ng/mL)	3.57 ± 1.35	3.30 ± 0.76	2.86 ± 0.57	2.74 ± 1.29	3.91 ± 0.73	0.178
QUICKI@	0.244 ± 0.009	0.245 ± 0.005	0.248 ± 0.006	0.252 ± 0.013	0.241 ± 0.005	0.126
Leptin (ng/mL)	26.99 ± 4.92	20.77 ± 6.90	17.73 ± 2.54	14.74 ± 3.67	34.34 ± 6.53	**0.000**
Body weight (g)	360.86 ± 7.97	344.71 ± 6.60	363.43 ± 10.85	360.86 ± 7.97	384.44 ± 7.41	**0.000**
Intrahepatic lipid#	18.24 ± 5.27	14.16 ± 5.31	19.02 ± 2.92	16.94 ± 4.84	29.06 ± 11.24	**0.003**
Intramyocellular lipids$	1.02 ± 0.28	1.07 ± 0.33	1.14 ± 0.27	1.21 ± 0.35	1.06 ± 0.19	0.755
SAT (mm^3^)	448.85 ± 179.71	290.80 ± 133.73	287.39 ± 61.14	207.55 ± 37.81	494.90 ± 113.62	**0.000**
VAT (mm^3^)	10410.44 ± 276.94	9333.13 ± 1103.97	9188.80 ± 627.74	8278.41 ± 1054.54	10610.23 ± 632.14	**0.000**
Daily food intake (g)	11.57 ± 0.51	9.64 ± 0.42	12.83 ± 1.05	12.77 ± 0.80	13.72 ± 0.47	**0.000**
Dietary calorie deficit* (kcal)	10.10	19.18	4.18	4.46	–	–

^#^Intrahepatic lipid was calculated by ratio of (Fat/(Fat + H_2_O))*100.

^$^Intramyocellular lipid = Intramyocellular lipid/total creatine.

^*^Dietary calorie deficits evaluated from daily food intake when compared to control.

^@^QUICKI = 1/(log(Fasting Glucose (mg/dL)) + log(Fasting Insulin (μU/mL)).

**Table 3:** Glass’s ∆ effect size of interventions with respect to the control, for serum metabolic parameters, body weight, adiposity, adipose depot volumes, ectopic fat and tibialis muscle weight and triglycerides (*small effect (∆ ≥ 0.2), **moderate effect (∆ ≥ 0.5), ***large effect (∆ ≥ 0.8)).

Measurement	Cohort			
	CR1(N = 7)	CR2(N = 7)	EX1(N = 7)	EX2(N = 7)
Triglycerides (mg/dL)	−0.24*	−0.86***	0.45*	1.61***
Glucose (mg/dL)	0.01	0.46*	0.61**	0.12
Insulin (ng/mL)	−0.47*	−0.84***	−1.44***	−1.60***
QUICKI@	0.63**	0.71**	1.38***	2.13***
Leptin (ng/mL)	−1.13***	−2.08***	−2.54***	−3.00***
Body weight (g)	−3.18***	−5.37***	−2.84***	−3.18***
% body fat	−1.16***	−2.73***	−3.82***	−5.62***
Intrahepatic lipid#	−0.96***	−1.33***	−0.89***	−1.08***
Intramyocellular lipids$	−0.19	0.07	0.41*	0.83***
SAT (mm^3^)	−0.41*	−1.80***	−1.83***	−2.53***
VAT (mm^3^)	−0.32*	−2.02***	−2.25***	−3.69***
Tibialis weight (mg)	−0.35*	0.15	1.62***	1.84***
Tibialis triglycerides (mg/g tissue)	−0.43*	−0.35*	−0.81***	−0.71**

^#^Intrahepatic lipid was calculated by ratio of (Fat/(Fat + H_2_O))*100.

^$^Intramyocellular lipid = Intramyocellular lipid/total creatine.

^@^QUICKI = 1/(log(Fasting Glucose (mg/dL)) + log(Fasting Insulin (μU/mL)).

**Table 4:** Glass’s ∆ effect size of interventions with respect to the control for muscle and liver desaturase indices (*small effect (∆ ≥ 0.2), **moderate effect (∆ ≥ 0.5), ***large effect (∆ ≥ 0.8)).

Measurement	Cohort			
	CR1(N = 7)	CR2(N = 7)	EX1(N = 7)	EX2(N = 7)
Muscle SCD16	−0.90***	−0.96***	−1.04***	−1.47***
Muscle SCD18	−0.91***	−0.44*	−0.67**	−1.10***
Muscle D6D	−0.96***	−1.18***	−1.47***	−1.39***
Muscle D5D	1.01***	1.49***	0.95***	1.82***
Liver SCD16	−0.04	−1.26***	−1.05***	−1.54***
Liver SCD18	0.03	−0.19	0.18	−0.03
Liver D6D	−0.68**	−1.01***	−2.28***	−2.37***
Liver D5D	1.33***	3.50***	3.06***	3.25***

**Table 5:** Glass’s ∆ effect size of interventions with respect to the control for muscle and liver acylcarnitines (*small effect (∆ ≥ 0.2), **moderate effect (∆ ≥ 0.5), ***large effect (∆ ≥ 0.8)).

Measurement	Cohort			
	CR1(N = 7)	CR2(N = 7)	EX1(N = 7)	EX2(N = 7)
Liver AC C0	0.72**	0.72**	0.51**	0.44*
Liver AC C2	−0.08	0.11	0.60**	−0.13
Liver AC C16:0	0.43*	−0.02	0.96***	0.33*
Liver AC C18:0	0.28*	−0.01	2.20***	1.11***
Liver AC C18:1	0.40*	−0.05	1.51***	0.68**
Muscle AC C0	0.77**	0.65**	1.20***	0.45*
Muscle AC C2	0.04	−0.25*	0.05	−0.56**
Muscle AC C4-OH	−0.45*	−0.60**	−0.20*	−1.00***
Muscle AC C16:0	0.42*	0.01	0.15	0.52**
Muscle AC C18:0	0.47*	0.30*	0.22*	0.72**
Muscle AC C18:1	0.40*	−0.01	0.21*	0.40*

**Table 6:** Association (Pearson r) between % body fat, insulin sensitivity, IHL, IMCL, adipose depots, muscle and liver acylcarnitines and muscle and liver desaturase indices obtained from the post-measurements in the pooled cohort (**P < 0.01 and *P < 0.05 in bold).

	% Body fat	QUICKI@	IHL	IMCL
Insulin sensitivity	−0.56^**^	**1**	−0.43^**^	0.17
Leptin	0.90^**^	−0.56^**^	0.68^**^	−0.23
SAT	0.72^**^	−0.32	0.48^**^	−0.18
VAT	0.84^**^	−0.52^**^	0.48^**^	−0.26
IHL	0.52^**^	−0.44^**^	1	−0.04
IMCL	−0.24	0.18	−0.04	1
Muscle AC C4-OH	0.32	−0.17	0.28	0.09
Muscle AC C0	0.02	0.06	−0.01	−0.15
Muscle AC C18:0	−0.11	0.11	−0.17	0.48^**^
Muscle AC C16:0	−0.03	0.08	−0.13	0.45^**^
Muscle AC C18:1	−0.01	0.05	−0.10	0.43^**^
Muscle SCD16	0.65^**^	−0.35^*^	0.44^**^	−0.29
Muscle D6D	0.56^**^	−0.44^**^	0.37^*^	−0.29
Muscle D5D	−0.64^**^	0.23	−0.54^**^	0.21
Liver SCD16	0.58^**^	−0.58^**^	0.61^**^	−0.26
Liver D6D	0.73^**^	−0.41	0.38	−0.32
Liver D5D	−0.73^**^	0.44^*^	−0.80^**^	0.08
Liver AC C0	0.12	0.12	−0.05	−0.14
Liver AC C16:0	−0.18	0.53^*^	−0.14	0.20
Liver AC C18:0	−0.38	0.65^**^	−0.14	0.27
Liver AC C18:1	−0.28	0.63^**^	−0.20	0.19

^@^QUICKI = 1/(log(Fasting Glucose (mg/dL)) + log(Fasting Insulin (μU/mL)).
